# KDDC: a new framework that integrates kmers, dataset filtering, dimension reduction and classification algorithms to achieve immune cell heterogeneity classification

**DOI:** 10.3389/fimmu.2025.1602907

**Published:** 2025-05-30

**Authors:** Nan Zhang, Shishun Zhao, Runze Wu, Xizi Luo, Ming Yang, Zecheng Chang, Jianting Xu

**Affiliations:** ^1^ Cancer Center, First Hospital of Jilin University, Changchun, China; ^2^ College of Mathematics, Jilin University, Changchun, China; ^3^ College of Basic Medical Science, Jilin University, Changchun, China

**Keywords:** KDDC, immune repertoire, cdr3aa, structural similarity, immune cell heterogeneity

## Abstract

**Introduction:**

Integrating immune repertoire sequencing data with single cell sequencing data offers profound insights into the diversity of immune cells and their dynamic changes across various disease states.

**Methods:**

Here, we propose a novel KDDC framework that integrates kmers, dataset selection, dimensionality reduction and classification algorithms to facilitate the heterogeneous classification of immune cells.

**Results and Discussion:**

By comparing various kmer length combinations across seven different classification algorithms, we found that B cell receptor-based cellsubset classification outperforms T cell receptor-based classification, achievingan average AUC of over 96%. This finding offers a new perspective on the classification of immune cells. We also observed that 11 distinct cell subpopulations exhibited differences in cell proportions, inflammatory factorexpression, cell communication, and metabolic pathways, with notable activity in metabolic pathways. These variations may reflect the adaptive changes of cellsubpopulations in response to different disease states. This study aims to uncoverthe potential biological significance of immune prediction, target antigens, andeffective evaluation by analyzing the immune characteristics of specific cellsubsets at the cellular level. These findings will not only enhance ourunderstanding of immune system functions but also offer new directions for the development and optimization of immunotherapy.

## 1 Introduction

After pathogens invade the human body, the immune system serves a crucial protective role, primarily through the immune response mediated by numerous lymphocyte receptors, including T cell receptors (TCRs) and B cell receptors (BCRs). Consequently, inferring the specific binding of immune cell receptors to antigens has emerged as a prominent research focus. The total count of unique TCR and BCR reflects the immune system’s capacity to respond to pathogen invasion (1, 2). Analyzing and annotating immune repertoire data can offer valuable insights for immune prediction, target antigens, and effective evaluation (3).

Each TCR and BCR is a dimer composed of two distinct chains. TCR are encoded by the cell’s neutralizing chain (4), while BCR consist of a heavy chain (IGH) and a light chain, with the light chain derived from either the κ (IGK) or λ (IGL) locus (5). Each antigen-specific receptor comprises variable (V), diversity (D), joining (J), and constant (C) gene segments (6). The combination of these gene segments determines the specificity and diversity of lymphocytes. TCR and BCR features three complementarity-determining regions (CDRs): CDR1, CDR2, and CDR3, with CDR3 being the most variable component of the antigen-binding site (7). The diversity and uniqueness of TCR and BCR enable their use as distinct molecular barcodes for T cells and B cells, facilitating the inference of antigen-specific response functions and elucidating the differences in the association between cell types and disease phenotypes. Moreover, the rapid advancement and application of single-cell sequencing technologies allow researchers to investigate the changes and mechanisms that occur in cells in response to pathogen invasion at the cellular level (8). Consequently, integrating single-cell sequencing with immune repertoire sequencing can provide a more comprehensive view of the transcriptional characteristics and immune behaviors of cells, aiding in the discovery of antibody variations among individuals and the changes in immune cells under disease conditions.

The research and application of immune repertoire data primarily encompass two areas. The first involves exploring VDJ gene rearrangement. For instance, Ren et al. utilized large-scale single-cell transcriptome maps to reveal the immune characteristics of COVID-19, employing BCR data to classify patients’ infection statuses based on a random forest classifier that analyzes VDJ gene usage frequency (9). Similarly, Zhao et al. proposed a learning-based machine learning model, VDJMiner, to automatically mine VDJ gene fragments from TCR data to predict COVID-19 prognosis (10). The second area focuses on structural similarity in research. For example, Shoukat et al. employed kmer-based principal component analysis and clustering algorithms to classify samples from COVID-19 patients using TCR sequencing data (11). Park et al. also utilized machine learning methods to identify TCR characteristics in COVID-19 patients (12). However, these studies predominantly analyze single BCR or TCR data and do not integrate BCR with TCR data to investigate the changes in patients across different infection states, nor do they examine the binding behaviors and mechanisms between cell types in single-cell sequencing.

In this study, we proposed a KDDC framework that achieves heterogeneous classification of immune cells through kmer cutting, dataset screening, dimensionality reduction, and the selection of various classification algorithms. We integrated single-cell sequencing data with immune repertoire data to identify the characteristics of patients’ immune repertoires through specific immune cell subsets. This approach enabled us to explore the binding behaviors and mechanisms of these cells. Our model also compared seven classification algorithms: Logistic Regression(LR), Support Vector Machine(SVM), Multi-layer Perceptron(MLP), K-Nearest Neighbors(KNN), Decision Tree(DC), Random Forest(RF) and eXtreme Gradient Boosting(XGBoost), to classify and evaluate infection status based on specific immune cell subsets. This study aims to uncover the potential biological significance for immune prediction, target antigens, and effective evaluation by analyzing the immune characteristics of specific cell subsets at the cellular level.

## 2 Materials and methods

### 2.1 Data collection and preprocessing

The data for this study come from two parts: the immune repertoire data is obtained through the GSE158055 dataset in the GEO database. The single-cell sequencing data is sourced from the website http://covid19.cancer-pku.cn/#/dimensional-reduction, which provides a complete h5 file containing B cell, CD4T cell and CD8T cell related data. Following data collection, the two datasets were matched, and we specially screened for B cells (BCRH_cdr3aa) and T cells (TCRA_cdr3aa) that include the cdr3 amino acid sequences for further analysis.

### 2.2 Cell clustering and cell annotation

We used the Scanpy library in Python for single-cell sequencing data analysis (13). The analysis proceed as follows: firstly, we performed K-Nearest Neighbor(KNN) clustering separately on the selected B cells and T cells to identify distinct cell subpopulations. After clustering, we identified the marker genes of each cell subpopulation based on the significant expression level. Using the identified marker genes, we employed manual annotation methods, supported by relevant literature, to classify the cell subpopulations. Finally, we visualized the cell annotation results using UMAP diagrams, which effectively illustrate the distribution and characteristics of the annotated cell subpopulations.

### 2.3 kmers clipping and dataset screening

The kmers algorithm segments each specific cdr3 amino acid sequence based on selected lengths. In this study, we chose k values of 2, 3 and 4. Based on these lengths, we combined the 20 common amino acid types to generate 400 amino acid concatemers for k=2, 8000 amino acid concatemers for k=3, and 160,000 amino acid concatemers for k=4. Next, we constructed a count matrix for CDR3 amino acid concatemers according to the different states of the same cell type, assembling the dataset based on the various k values. This process resulted in seven datasets containing CDR3 amino acid concatemers. During the construction of the count matrix, any concatemers that did not appear were filled with 0 to ensure accurate counting.

### 2.4 Choices of dimensionality reduction and classification methods

Given the large number of features in the dataset, we first counted the number of filled 0 values in each column and set a threshold to eliminate features containing outliers.

The threshold filtering mainly considered the expression of feature in the sample, which determined whether to retain the features. Next, we employed analysis of variance (ANOVA) for feature dimensionality reduction, retaining only those features with a significance level lower than 0.05. After dimensionality reduction, we divided each dataset into a training dataset and a test dataset in a 4:1, using seven different classification algorithms to classify and compare each cell type based on structural similarity. The algorithms included: LR, SVM, MLP, KNN, DC, RF and XGB. In the final presentation, we selected the classification algorithm that exhibited the best performance. Furthermore, the grid search algorithm was applied to introduce hyperparameters to filter out the best classification model, with a p-value range of 0.001-0.049. Comparing multiple classification models through changes in p-value to find the best combination and improve the generalization ability. To address the imbalance in data distribution, we utilized the ROC curve and the average AUC value as evaluation indicators for the classification results. The advantage of ROC curve was that it was intensive to data imbalance and effectively demonstrate the classification performance across the entire threshold range. The average macro AUC was calculated independently for each category, avoiding the influence of large categories and not relying on the number of categories (14, 15).

### 2.5 Connections between cell subpopulations and metabolic pathway score

To explore the connections between cell subpopulations, we measured the interaction between them based on the membrane protein pairs present on the surface of the cells in different states (16). This assessment provided insights into the interactions among cell subpopulations. For calculating the metabolic pathway scores of different cell subpopulations, we accessed the KEGG database (KEGG, https://www.kegg.jp/) and utilized the KEGGREST package in R to screen 84 human-related metabolic pathways (17). We then combined the expression levels of metabolic genes associated with each pathway to compute a score for each pathway in various states. Additionally, we conducted significance testing for each metabolic pathway to highlight the differences among cell subpopulations under different states. The t test method was used for pairwise comparison, while the Kruskal Wallis test method was used for multiple comparison. This approach allowed us to identify key metabolic pathways that may be relevant to the functional characteristics of the cell subpopulations in response to different conditions.

## 3 Results

### 3.1 KDDC framework: classification of cell heterogeneity based on amino acid sequence similarity

**Figure 1:**
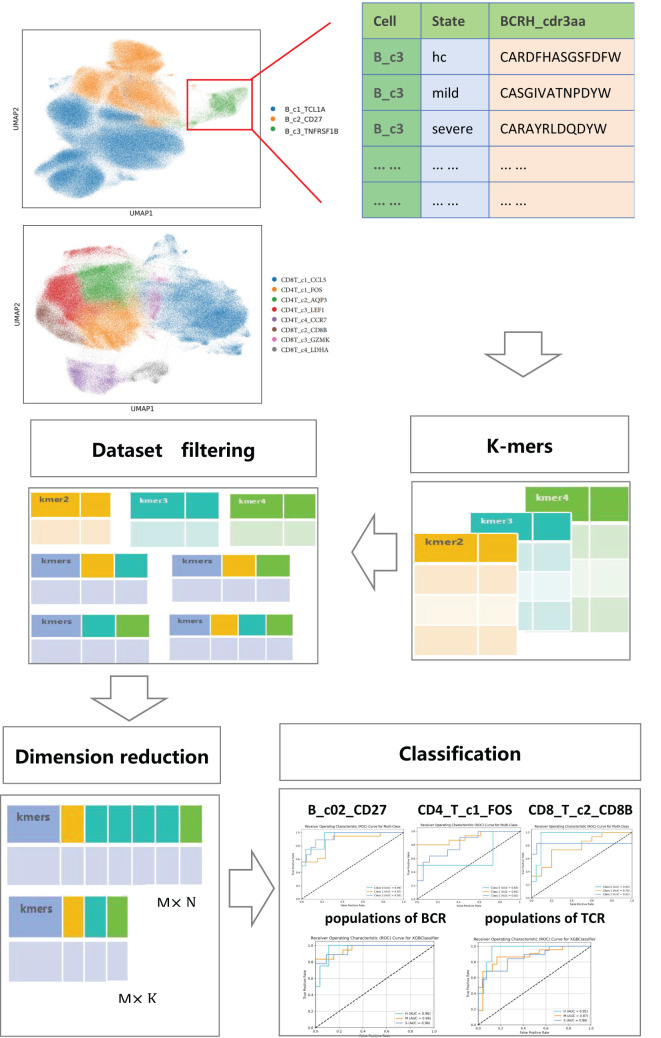
A summary diagram of the KDDC framework.

We propose a novel framework that integrates k-mer cutting, dataset screening, dimensionality reduction and classification algorithms for a joint analysis of immune repertoire data and single-cell sequencing data. This framework achieves heterogeneous classification of immune cells based on the structural similarity of CDR3 amino acid sequences, providing new insights for personalized disease treatment. The framework consists of five main parts: the first part realizes cell clustering and cell annotation through single-cell sequencing data, and then uses the immune repertoire data to extract the cdr3 amino acid sequences of the same cell type under each cell type and construct the corresponding dataset. The second part is to perform kmers processing on the cdr3 amino acid sequences in the dataset to cut out amino acid concatemers of different kmer lengths. The third part is to generate the corresponding count matrix for the amino acid concatemers according to different cutting lengths. The fourth part is to reduce the dimension of the count matrix of the amino acid concatemers using feature selection methods. The fifth part is to classify and compare the count matrix after dimensionality reduction using seven different classification algorithms to achieve heterogeneous classification of immune cell subsets. A schematic diagram of the framework is provided in **Figure 1**, illustrating the workflow and interconnections among these components.

### 3.2 Demonstration of immune cell subset distribution in COVID-19 patients based on single-cell sequencing data

**Figure 2:**
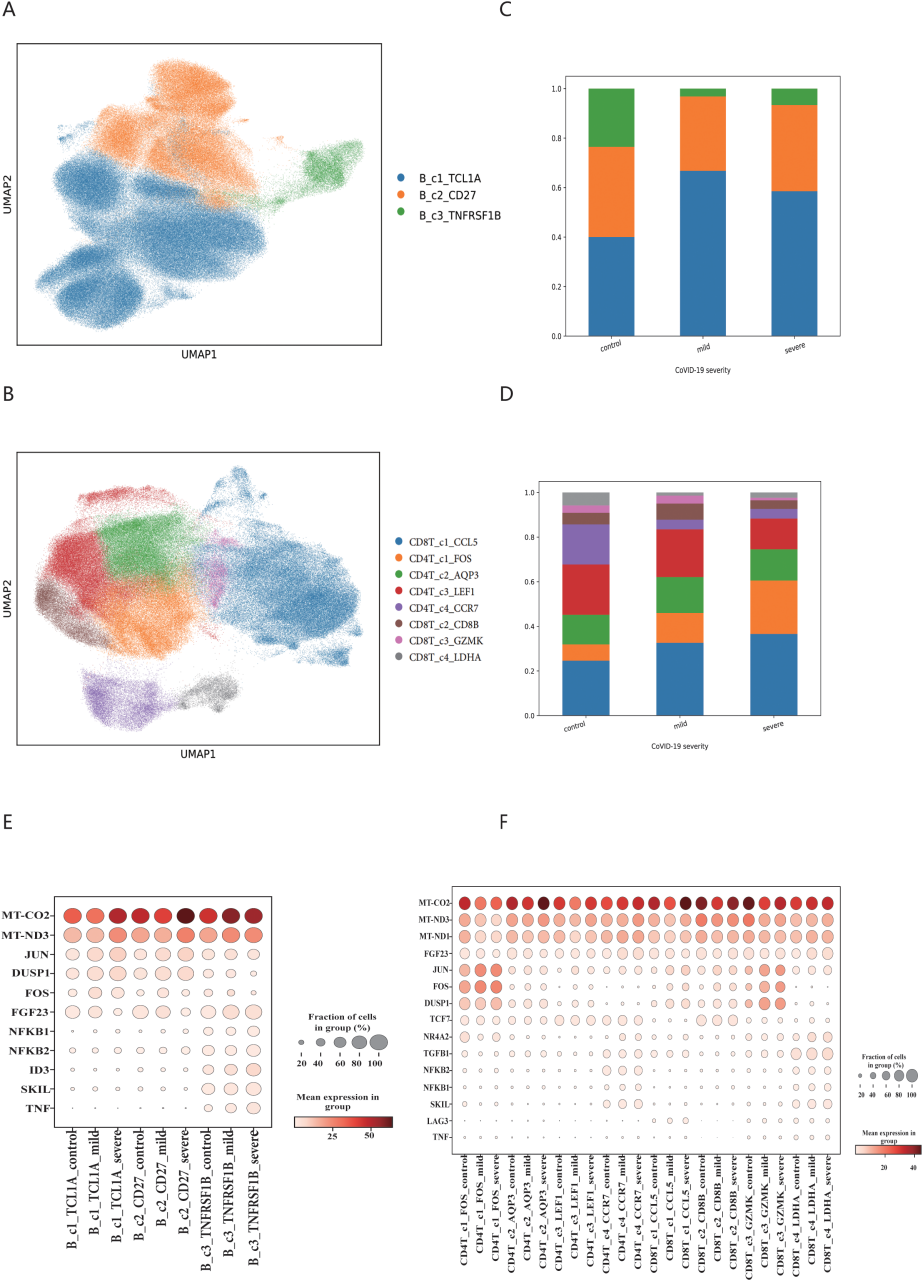
The immune cell populations signatures in COVID-19 patient. **(A)** Map of B cell subsets in COVID-19 patients based on BCR data. **(B)** Map of T cell subsets in COVID-19 patients based on TCR data. **(C)** Changes in the proportion of B cell subsets. **(D)** Changes in the proportion of T cell subsets. The legend of C and D should be consistent with A and B respectively. **(E)** Differential expression of inflammatory factors in B cell subsets. **(F)** Differential expression of inflammatory factors in T cell subsets.

We investigated the shared cells between the immune repertoire data and single-cell sequencing data, ultimately identifying 278,298 B cells and 220,968 T cells, as illustrated in **Supplementary Figure 1**. Using single-cell sequencing data, we generated B cell and T cell profiles of COVID-19 patients, as shown in **Figure 2A** and **Figure 2B**. For B cells, we identified three cell subpopulations, with the marker genes of each cell subpopulation displayed in **Supplementary Figures 2A–C**. We found that the proportion of B_c1_TCL1A cell subpopulation increased significantly after COVID-19 infection, and the proportion of B_c3_TNFRSF1B subpopulation cells decreased significantly, as shown in **Figure 2C**. For T cells, we identified eight cell subpopulations, and the marker genes for each cell subpopulation are shown in **Supplementary Figures 2D–K**. We found that the proportion of CD4T_c1_FOS and CD8T_c4_LDHA cell subpopulation increased after COVID-19 infection. The proportion of CD4T_c4_CCR7 and CD8T_c1_CCL5 cell subpopulation shown a notable decrease, as shown in **Figure 2D**. We examined the inflammatory factors related to immune cells and discovered that the expression of MT-CO2 and MT-ND3 genes was significantly elevated in both B cell and T cell subsets following COVID-19 infection. The B_c3_TNFRSF1B populations shown a significant increase in the expression of NFKB1, NFKB2, ID3, SKIL and TNF genes. Conversely, B_c1_TCL1A, CD4T_c1FOS and CD8T_c3_GZMK subpopulations exhibited overexpression of DUSP1 and JUN genes. This result displayed in **Figures 2E, F**.

### 3.3 The KDDC framework identifies heterogeneous classification of cell subsets in B cells

**Figure 3:**
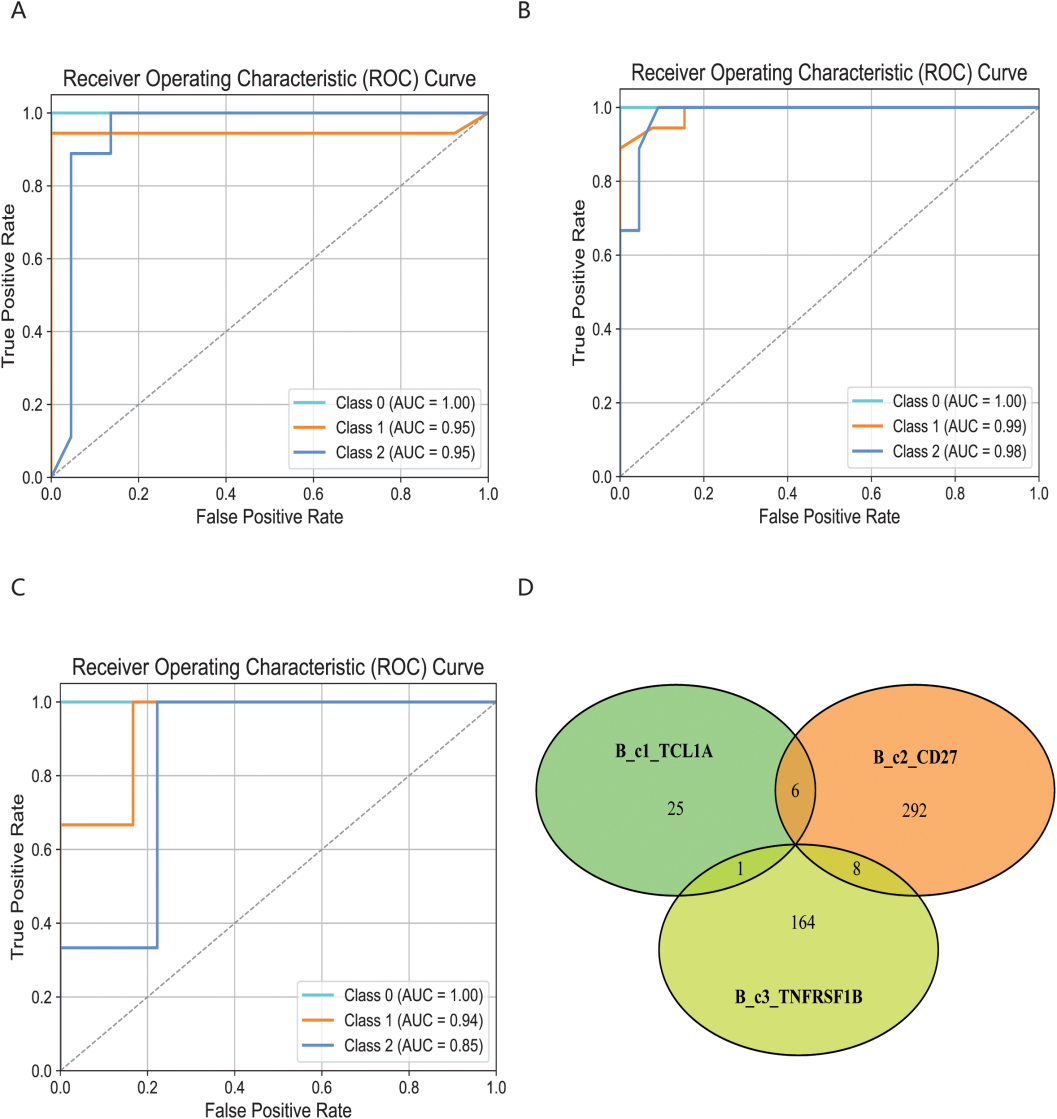
Comparison of specific B cell subsets based on structural similarities. **(A)** Classification and comparison of B_c1_TCL1A cell subsets based on structural similarity. **(B)** Classification and comparison of B_c2_CD27 cell subsets based on structural similarity. **(C)** Classification and comparison of B_c3_TNFRSF1B cell subsets based on structural similarity. The number of class 0, 1 and 2 are normal group, mild and severe infection, respectively. **(D)** Interactive analysis of structural features for optimal classification of three specific cell subpopulations.

To characterize the structure of cdr3 amino acid sequences among different immune cell subsets, we applied the KDDC framework to achieve heterogeneous classification of immune cells. We constructed a count matrix using cdr3 sequence amino acid concatemers of varying lengths and compared the classification results to reflect the heterogeneity of different immune cell subsets. Using variance analysis, we performed dimensionality reduction on the count matrix and evaluated several classification algorithms for comparison. ultimately, we selected the most suitable classification algorithm for our study. The classification results of each algorithm are shown in **Table 1**. Our findings indicate that the classification performance for the three B cell subsets was notably strong. Specifically, the average AUC value for the B_c1_TCL1A cell subset using kmer3 reached 96.5%, as shown in **Figure 3A**. The average AUC value for the B_c2_CD27 cell subset across kmer2, kmer3 and kmer4 reached an impressive 99%, depicted in **Figure 3B**. Additionally, the average AUC value for the B_c3_TNFRSF1B cell subset with kmer2, kmer3 and kmer4 reached 93.2%, illustrated in **Figure 3C**. The relevant parameters of the best model can refer to **Supplementary Table 1**. Afterwards, we conducted an interactive analysis on the three kmers datasets that demonstrated the best classification results. We discovered share amino acid concatemers among the kmers dataset, which reflect the structural similiarities with cdr3 sequences. The structure of DLW and FDL were high importance in B_c1_TCL1A and B_c2_CD27. Meanwhile, the structure of YDY and RGGF were great importance in B_c2_CD27 and B_c3_TNFRSF1B, as displayed in **Figure 3D**.

**Table 1:** B cell subset classification and assessment based on BCR sequencing.

BCR	kmer	Model	macro_AUC	p-value	H	M	S
B_c1_TCL1A	kmer2	RF	0.879	0.041	0.935	0.855	0.848
	kmer3	RF	0.965	0.006	1	0.947	0.947
	kmer4	XGB	0.933	0.046	0.88	0.918	1
	kmer2_3	RF	0.96	0.012	0.995	0.934	0.95
	kmer2_4	RF	0.924	0.031	0.977	0.889	0.907
	kmer3_4	RF	0.962	0.025	1	0.932	0.955
	kmer2_3_4	RF	0.958	0.006	0.991	0.925	0.957
B_c2_CD27	kmer2	RF	0.938	0.048	1	0.929	0.886
	kmer3	RF	0.97	0.045	1	0.927	0.982
	kmer4	RF	0.935	0.047	0.92	0.944	0.94
	kmer2_3	RF	0.978	0.012	1	0.934	1
	kmer2_4	RF	0.977	0.037	0.981	0.987	0.962
	kmer3_4	MLP	0.986	0.045	1	0.957	1
	kmer2_3_4	RF	0.99	0.046	1	0.989	0.982
B_c3_TNFRSF1B	kmer2	RF	0.593	0.05	0.667	0.667	0.444
	kmer3	RF	0.889	0.028	0.87	0.889	0.907
	kmer4	RF	0.881	0.033	0.829	1	0.813
	kmer2_3	RF	0.926	0.04	0.981	0.944	0.852
	kmer2_4	RF	0.923	0.018	0.926	0.917	0.926
	kmer3_4	RF	0.889	0.025	0.87	0.889	0.907
	kmer2_3_4	RF	0.932	0.039	1	0.945	0.852

### 3.4 KDDC framework identifies heterogeneous classification of cell subsets in T cells

**Figure 4:**
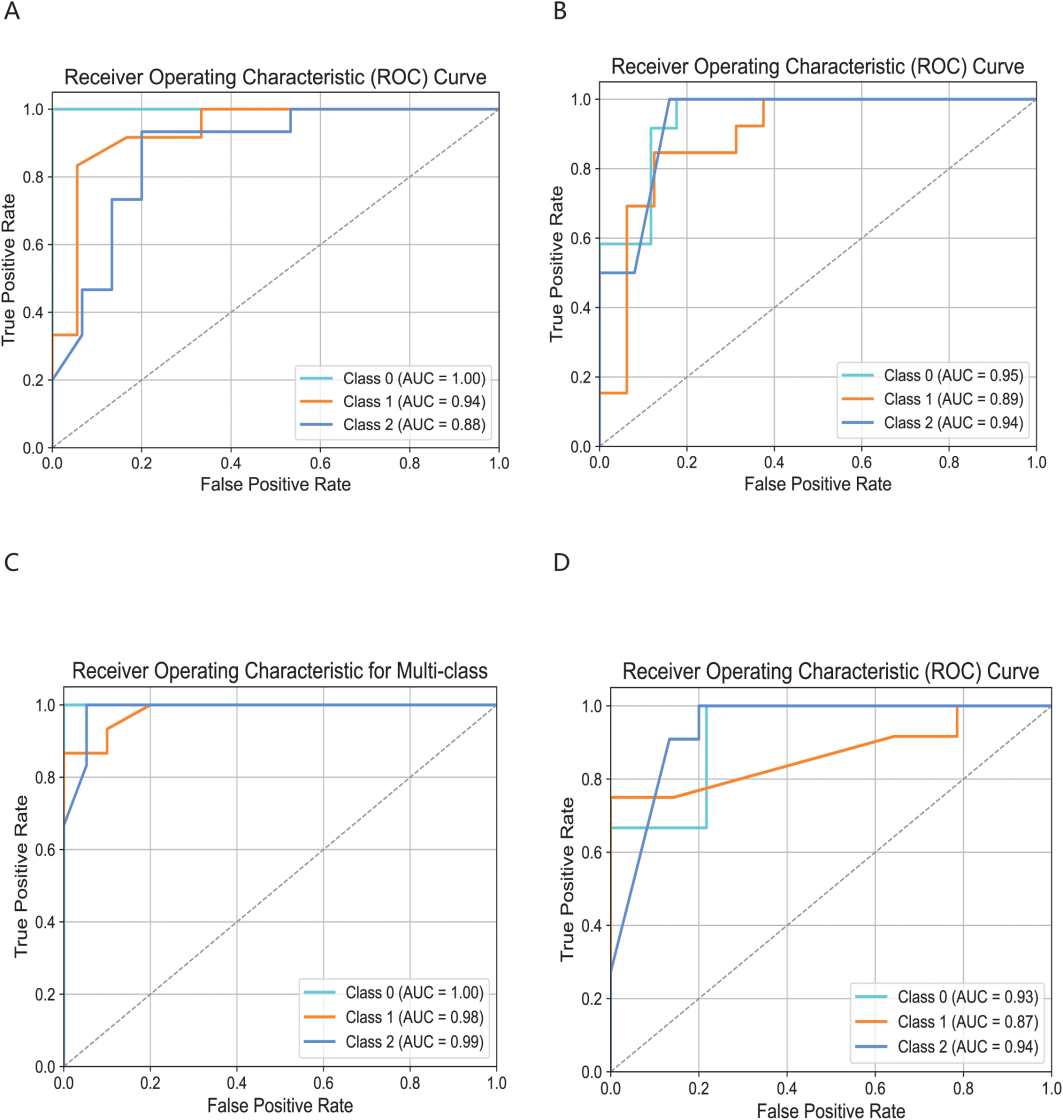
Comparison of specific T cell subsets based on structural similarities. **(A)** Classification and comparison of CD4T_c2_AQP3 cell subsets based on structural similarity. **(B)** Classification and comparison of CD4T_c3_LEF1 cell subsets based on structural similarity. **(C)** Classification and comparison of CD8T_c2_CD8B cell subsets based on structural similarity. **(D)** Classification and comparison of CD8T_c3_GZMK cell subsets based on structural similarity. The number of class 0, 1 and 2 are normal group, mild and severe infection, respectively.

The cell subsets within T cells were classified and compared using the KDDC framework, with the results of different classification algorithms summarized in **Tables 2** and **3**. We discovered that the CD4T_c2_AQP3 subset had a strong classification performance in kmer4, achieving an average AUC value of 93.5%, as illustrated in **Figure 4A**. The CD4T_c3_LEF1 subset demonstrated effective classification with kmer4, yielding an average AUC value of 92.7%, as shown in **Figure 4B**. The classification performance for the CD8T_c2_CD8B subset was optimized using kmer2, kmer3 and kmer4, attaining an impressive average AUC value of 99%, represented in **Figure 4C**. The CD8T_c3_GZMK subset exhibited a better classification performance in kmer2 and kmer3, resulted in an average AUC value of 91.2%, depicted in **Figure 4D**.

**Table 2:** Evaluation of CD4T cell subsets based on TCR sequencing.

CD4T	kmer	Model	macro_AUC	p-value	H	M	S
CD4T_c1_FOS	kmer2	MLP	0.848	0.018	0.856	0.841	0.846
	kmer3	DC	0.818	0.036	0.706	0.767	0.981
	kmer4	RF	0.861	0.015	0.981	0.765	0.838
	kmer2_3	RF	0.839	0.03	0.781	0.756	0.981
	kmer2_4	XGB	0.826	0.017	0.799	0.756	0.923
	kmer3_4	RF	0.827	0.03	0.754	0.767	0.962
	kmer2_3_4	MLP	0.831	0.018	0.77	0.779	0.942
CD4T_c2_AQP3	kmer2	RF	0.608	0.049	0.486	0.686	0.652
	kmer3	MLP	0.847	0.03	0.791	0.769	0.981
	kmer4	MLP	0.937	0.026	1	0.935	0.876
	kmer2_3	MLP	0.784	0.021	0.684	0.774	0.893
	kmer2_4	XGB	0.801	0.003	0.667	0.735	1
	kmer3_4	KNN	0.832	0.035	0.852	0.824	0.821
	kmer2_3_4	KNN	0.797	0.032	0.739	0.724	0.929
CD4T_c3_LEF1	kmer2	DC	0.839	0.031	0.757	0.762	1
	kmer3	RF	0.903	0.017	0.876	0.869	0.965
	kmer4	RF	0.927	0.02	0.94	0.894	0.946
	kmer2_3	RF	0.875	0.046	0.867	0.8	0.96
	kmer2_4	MLP	0.875	0.041	0.899	0.767	0.96
	kmer3_4	RF	0.889	0.004	0.879	0.898	0.89
	kmer2_3_4	RF	0.891	0.004	0.909	0.855	0.91
CD4T_c4_CCR7	kmer2	LR	0.854	0.049	0.889	0.81	0.864
	kmer3	SVM	0.733	0.049	0.722	0.476	1
	kmer4	RF	0.706	0.049	0.625	0.867	0.625
	kmer2_3	RF	0.575	0.049	0.444	0.69	0.591
	kmer2_4	MLP	0.868	0.049	0.889	0.714	1
	kmer3_4	SVM	0.788	0.049	0.722	0.643	1
	kmer2_3_4	MLP	0.868	0.049	0.889	0.714	1

**Table 3:** Evaluation of CD8T cell subsets based on TCR sequencing.

CD8T	kmer	Model	macro_AUC	p-value	H	M	S
CD8_T_c1_CCL5	kmer2	MLP	0.818	0.049	0.731	0.794	0.929
	kmer3	RF	0.843	0.007	0.761	0.779	0.988
	kmer4	DC	0.853	0.041	0.95	0.819	0.79
	kmer2_3	RF	0.839	0.03	0.781	0.756	0.981
	kmer2_4	MLP	0.778	0.015	0.63	0.739	0.964
	kmer3_4	RF	0.782	0.039	0.67	0.653	0.994
	kmer2_3_4	LR	0.7801	0.047	0.761	0.723	0.857
CD8_T_c2_CD8B	kmer2	RF	0.846	0.049	0.829	0.87	0.839
	kmer3	SVM	0.936	0.031	0.912	0.907	0.988
	kmer4	MLP	0.96	0.03	0.912	0.967	1
	kmer2_3	SVM	0.972	0.024	0.978	0.937	1
	kmer2_4	MLP	0.98	0.034	0.974	0.967	1
	kmer3_4	MLP	0.955	0.042	0.912	0.953	1
	kmer2_3_4	SVM	0.99	0.015	0.987	0.983	1
CD8_T_c3_GZMK	kmer2	SVM	0.766	0.049	0.83	0.744	0.725
	kmer3	MLP	0.87	0.012	0.897	0.786	0.928
	kmer4	SVM	0.861	0.045	0.806	0.833	0.942
	kmer2_3	RF	0.912	0.012	0.928	0.869	0.939
	kmer2_4	RF	0.879	0.012	0.909	0.821	0.906
	kmer3_4	MLP	0.865	0.019	0.815	0.824	0.956
	kmer2_3_4	RF	0.888	0.012	0.927	0.839	0.899
CD8_T_c4_LDHA	kmer2	RF	0.745	0.049	0.833	0.7	0.7
	kmer3	RF	0.622	0.049	0.667	0.6	0.6
	kmer4	RF	0.622	0.049	0.667	0.6	0.6
	kmer2_3	RF	0.744	0.049	0.833	0.7	0.7
	kmer2_4	RF	0.744	0.049	0.833	0.7	0.7
	kmer3_4	RF	0.622	0.049	0.667	0.6	0.6
	kmer2_3_4	RF	0.744	0.049	0.833	0.7	0.7

### 3.5 Close connections between immune cell subsets in different infection states in COVID-19 patients

**Figure 5:**
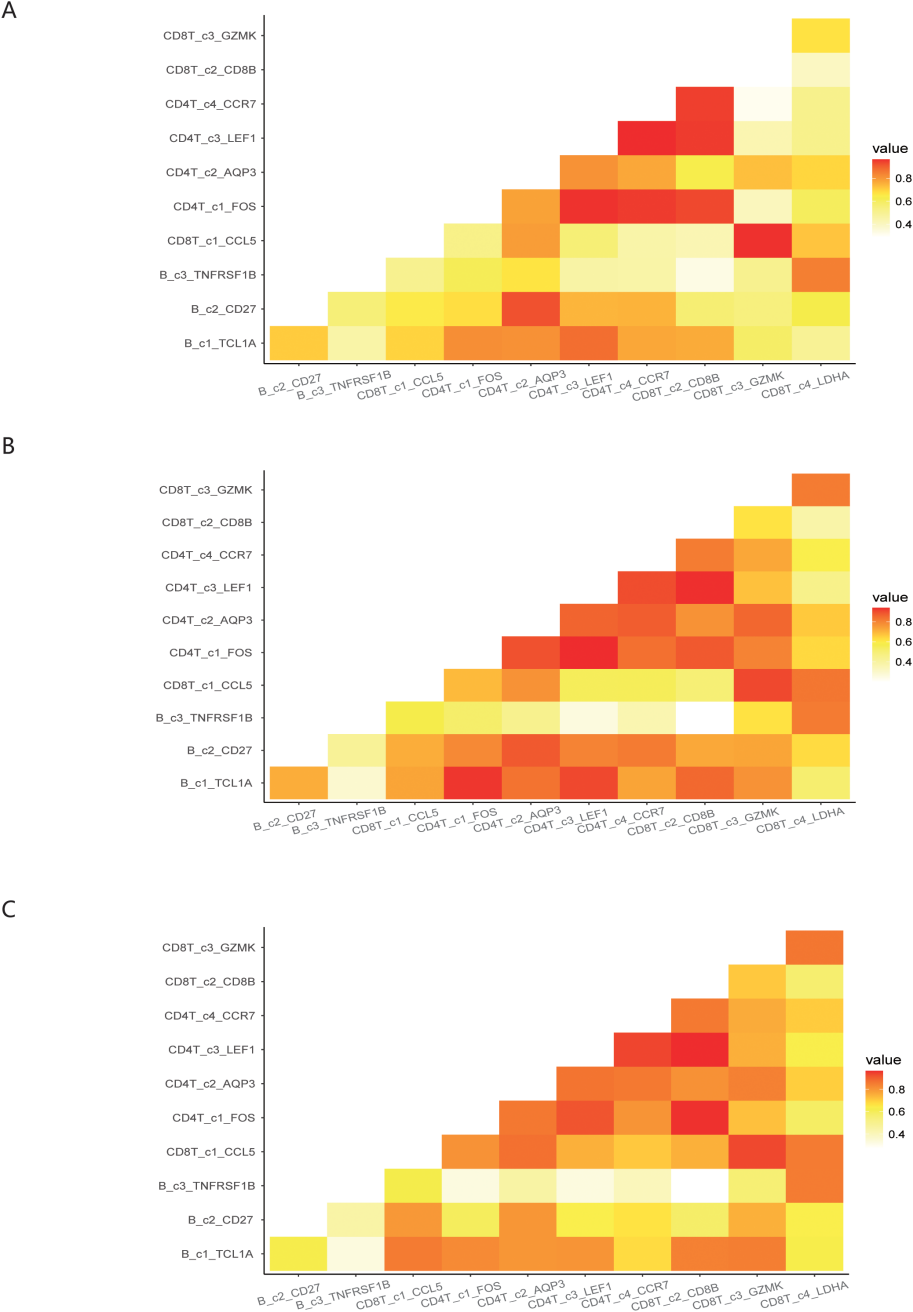
Demonstration of interactions between immune cell subsets under different infection states. **(A)** Interactions among immune cell subsets in normal states of COVID-19 patients. **(B)** Interactions among immune cell subsets in mild states of COVID-19 patients. **(C)** Interactions among immune cell subsets in severe states of COVID-19 patients.

We investigated the interactions among 11 immune cell subsets by selecting immune cell surface protein pairs. This approach allowed us to evaluate the connectivity of the immune cell subsets in COVID-19 patients under different infection states, based on the affinity of each pair of proteins and the expression of the encoding genes. When comparing the post-COVID-19 infection state to the normal state, we observed a weakened binding affinity between the CD4T_c4_CCR7 and CD8T_c2_CD8B subset. Conversely, the binding strength of CD8T_c1_CCL5 to both CD8T_c4_LDHA and CD4T_c1_FOS was found to be enhanced. In comparisons between the mid-term and severe states following COVID-19 infection, we noted a significant reduction in the binding affinity between the B_c1_TCL1A and CD4T_c1_FOS subset, as illustrated in **Figures 5A–C**.

### 3.6 Metabolic changes of immune cell subsets in COVID-19 patients under different infection states

**Figure 6:**
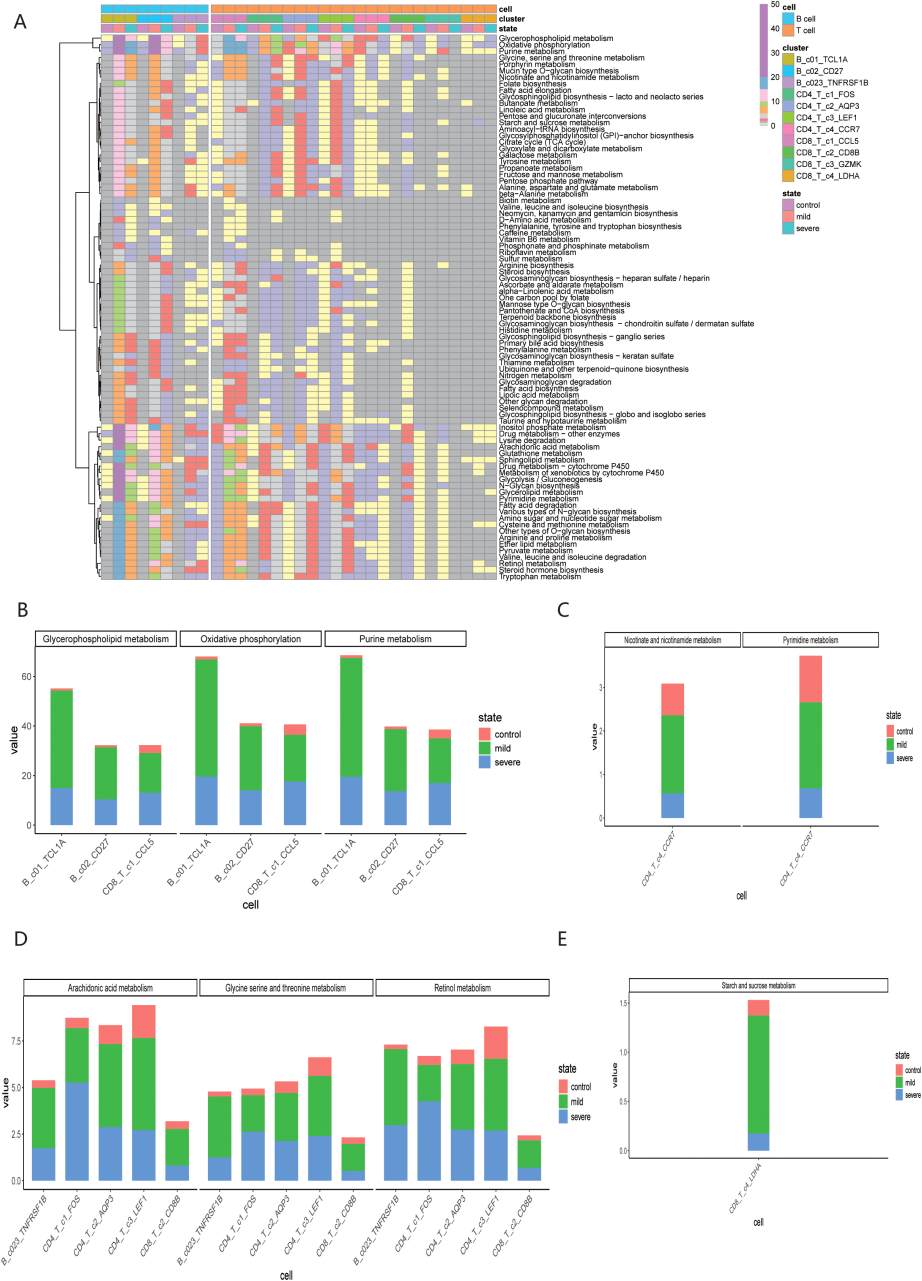
Metabolic heterogeneity of immune cells under different infection states. **(A)** Metabolic pathway scores of 11 immune cell subsets; **(B)** Significant expression of B_c1_TCL1A, B_c2_CD27 and CD8T_c1_CCL5 in three specific metabolic pathways. **(C)** Significant expression of B_c3_TNFRAF1B, CD4T_c1_FOS, CD4T_c2_AQP3, CD4T_c3_LEF1 and CD8T_c2_CD8B in three specific metabolic pathways. **(D)** Significant expression of CD4T_c4_CCR7 in two specific metabolic pathways. **(E)** Significant expression of CD8T_c4_LDHA in the specific metabolic pathway.

Furthermore, we explored the specific metabolic pathways associated with the 11 immune cell subpopulations. By comparing the post-COVID-19 infection state with the normal state, we observed that these cell subpopulations exhibited active engagement in various metabolic pathways, revealing metabolic heterogeneity across different infection states, as shown in **Figure 6A**. Meanwhile, we also conducted significant hypothesis tests on the metabolic pathways involved, as shown in the **Supplementary Figure 3**. The Glycerophospholipid metabolism, Oxidative phosphorylation, and Purine metabolism metabolic pathways were significantly expressed in B_c1_TCL1A, B_c2_CD27, and CD8T_c1_CCL5 cell subpopulations, particularly notable during the mid-stage of infection, as illustrated in **Figure 6B**. The CD4T_c4_CCR7 subpopulation shown significant changes in the scores of Pyrimidine metabolism, Nicotinate and nicotinamide metabolism metabolic pathways post-COVID-19 infection, as followed in **Figure 6C**. Additionally, the metabolic scores for the B_c3_TNFRSF1B, CD4T_c2_AQP3, CD4T_c3_LEF1, CD8T_c2_CD8B, and CD8T_c3_GZMK subpopulations displayed a trend of increasing followed by a decrease, as summarized in **Figure 6D**. The CD8T_C4_LDHA subpopulation exhibited notable changes in the scores for the Starch and sucrose metabolism metabolic pathways following COVID-19 infection, as summarized in **Figure 6E**.

### 3.7 The KDDC framework can also be used to classify COVID-19 patients in different infection states

**Figure 7:**
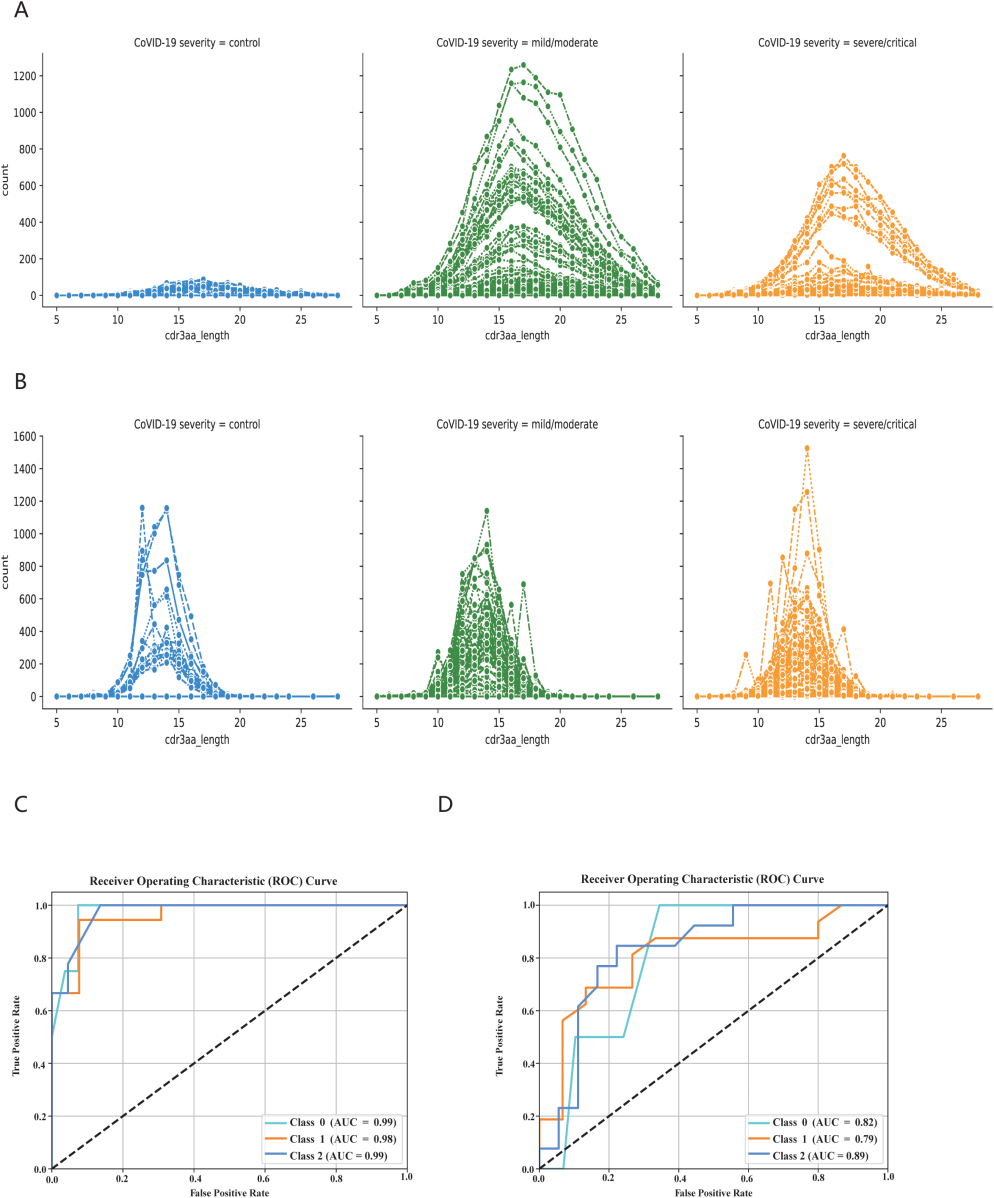
KDDC framework realizes classification and comparison of COVID-19 patients in different states. **(A)** BCR length distribution in different states of COVID-19 patients. **(B)** TCR length distribution in different states of COVID-19 patients. Different colors represent different states of patients, with blue indicating normal group, green mild or moderate infection, orange indicating severe infection. **(C)** Classification comparison of COVID-19 patients in different states based on BCR data. **(D)** Classification comparison of COVID-19 patients in different states based on TCR data.

Finally, we employed the KDDC algorithm to classify COVID-19 patients in different states based on BCR and TCR sequencing data. We first analyzed the distribution of cdr3aa sequence lengths in both BCR and TCR across different infection states. The cdr3aa length distribution exhibited significant differences post-COIVD-19 infection, as depicted in **Figures 7A**, **B**. The KDDC framework was applied to compare datasets with different kmer lengths. We identified the datasets and classification algorithms yielded the best classification performance, as summarized in **Table 4**. The classification performance of COVID-19 patients based on BCR sequencing data was exceptional, achieving an average AUC value of 98.8% in kmer3, as shown in **Figure 7C**. In contrast, the classification performance of COVID-19 patients based on TCR sequencing data was good, with an average AUC value of 83.3% in kmer4, as followed in **Figure 7D**.

**Table 4:** Evaluation of immune repertoire data classification algorithms.

IR	kmer	Model	macro_AUC	p-value	H	M	S
BCR	kmer2	RF	0.956	0.049	0.972	0.925	0.97
	kmer3	RF	0.988	0.034	0.991	0.983	0.99
	kmer4	RF	0.942	0.004	0.924	0.929	0.972
	kmer2_3	RF	0.98	0.014	0.981	0.968	0.992
	kmer2_4	RF	0.978	0.01	0.991	0.981	0.962
	kmer3_4	RF	0.984	0.039	1	0.981	0.972
	kmer2_3_4	RF	0.986	0.024	1	0.972	0.985
TCR	kmer2	XGB	0.751	0.045	0.905	0.668	0.681
	kmer3	XGB	0.797	0.006	0.929	0.752	0.71
	kmer4	RF	0.833	0.048	0.818	0.794	0.888
	kmer2_3	XGB	0.832	0.049	0.917	0.794	0.786
	kmer2_4	RF	0.782	0.004	0.964	0.671	0.712
	kmer3_4	XGB	0.81	0.047	0.905	0.752	0.773
	kmer2_3_4	XGB	0.813	0.04	0.905	0.769	0.765

## 4 Discussion

In this study, we implemented the KDDC framework to integrate single-cell sequencing data with immune repertoire data, achieving heterogeneous classification of immune cell subsets based on the structural similarity of cdr3aa sequences. The integration of immune repertoire data and single-cell sequencing data enhance our understanding of the changes, behaviors and mechanisms of cell subsets at the cellular level. We observed that the classification performance of BCR-based cell subsets under different infection states was notably superior to that of TCR-based cell subsets. This highlights the advantages of utilizing BCR data in understanding immune responses. Additionally, we placed emphasis on comparing the classification performance of BCR and TCR across individual cases, providing valuable insights into the distinct roles these receptors play in immune classification.

After COVID-19 infects the host, the expression of BCR and TCR receptor-related cell subpopulations shows heightened activity in terms of cell proportions, cell communication, and metabolic pathways. We observed a increase in the expression of MT-CO2 and MT-ND3 genes in both B cells and T cell subsets post-COVID-19 infection. This upregulation may activate oxidative phosphorylation and other related pathways, triggering immune responses (18). The B_c1_TCL1A subpopulation exhibited distinct changes in the cell proportions compared to the B_c3_TNFRSF1B subpopulation. The B_c3_TNFRSF1B subpopulation shown a marked increase in the expression of NFKB1, NFKB3, ID3, SKIL and TNF genes, potentially activating TGF-beta signaling pathway, which may contribute to cell proliferation (19). Conversely, the B_c1_TCL1A, CD4T_c1FOS and CD8T_c3_GZMK subpopulations were highly expressed in DUSP1 and JUN genes, suggesting activation the innate immune system and inflammatory response pathways (20). This may influence the production and expression of the corresponding cell subpopulations. Comparing the states before and after COVID-19 infection revealed a weakening of binding affinity between the CD4T_c4_CCR7 and CD8T_c2_CD8B subpopulations. In contrast, the binding strength between the CD8T_c1_CCL5, CD8T_c4_LDHA and CD4T_c1_FOS populations increased post-COVID-19 infection. These findings highlighted the complex interplay of immune cell subsets during COVID-19 infection, emphasizing the dynamic changes in gene expression and cell communication that contribute to the overall immune response.

During the metabolic process after COVID-19 infection, the B_c1_TCL1A, B_c2_CD27, and CD8T_c1_CCL5 subpopulations shown significant expression in the Glycerophospholipid metabolism, Oxidative phosphorylation, and Purine metabolism metabolic pathways, particularly during the middle stage of infection. Glycerophospholipid metabolism played a crucial role in maintaining the fluidity and integrity of cell membranes and was involved in inflammatory and immune responses during signal transduction (21). The increase in oxidative phosphorylation indicated that immune cells require substantial energy to support heightened activity during the immune response (22). Alterations in purine metabolism may drive cell proliferation and apoptosis, impacting overall cell function and survival (23). the CD4T_c1_FOS cell subpopulation exhibited significant increases in Retinol metabolism, Glycine, serine and threonine metabolism, and Arachidonic acid metabolism metabolic pathways after COVID-19 infection. The scores of these pathways for the B_c3_TNFRSF1B, CD4T_c2_AQP3, CD4T_c3_LEF1, CD8T_c2_CD8B and CD8T_c3_GZMK subpopulations displayed a trend of increasing following by a decreased. The Retinol metabolism was involved in the development and function of B cells and T cells and had anti-inflammatory properties (24). The Glycine, serine and threonine metabolism reflected the metabolic activity and energy demands of cells (25). The Arachidonic acid metabolism played a vital role in inflammatory responses, with its upregulation likely linked to enhanced immune regulation post-COVID-19 infection (26). The CD4T_c4_CCR7 subpopulation demonstrated significant shifts in the Pyrimidine metabolism, Nicotinate and nicotinamide metabolism metabolic pathways, potentially related to viral immune evasion mechanism and the metabolic reprogramming. The CD8T:C4_LDHA subpopulation shown notable changes in the Starch and sucrose metabolism pathway, suggesting adaptations in energy supply and the regulation of immune responses (27). Cell therapy based on TCR and BCR had significant advantages in immunotherapy, as it can accurately target immune cells, avoid damaging normal cells and improve treatment efficacy. This study provides valuable insights into the integrated analysis of immune repertoire data and single cell repertoire data. By revealing the metabolic changes and mechanisms of cell subpopulations at a cellular level, it offered new directions for personalized treatment strategies and antibody development in the context of COVID-19.

## Data Availability

The original contributions presented in the study are included in the article/**Supplementary Material**. Further inquiries can be directed to the corresponding authors.
