# Supplementary material for: KDDC: a new framework that integrates kmers, dataset filtering, dimension reduction and classification algorithms to achieve immune cell heterogeneity classification
Source: Front Immunol. 2025 May 30;16:1602907. doi: 10.3389/fimmu.2025.1602907 (PMC12162500; doi:10.3389/fimmu.2025.1602907)
Supplement: Supplementary file 1 [file DataSheet1.docx]

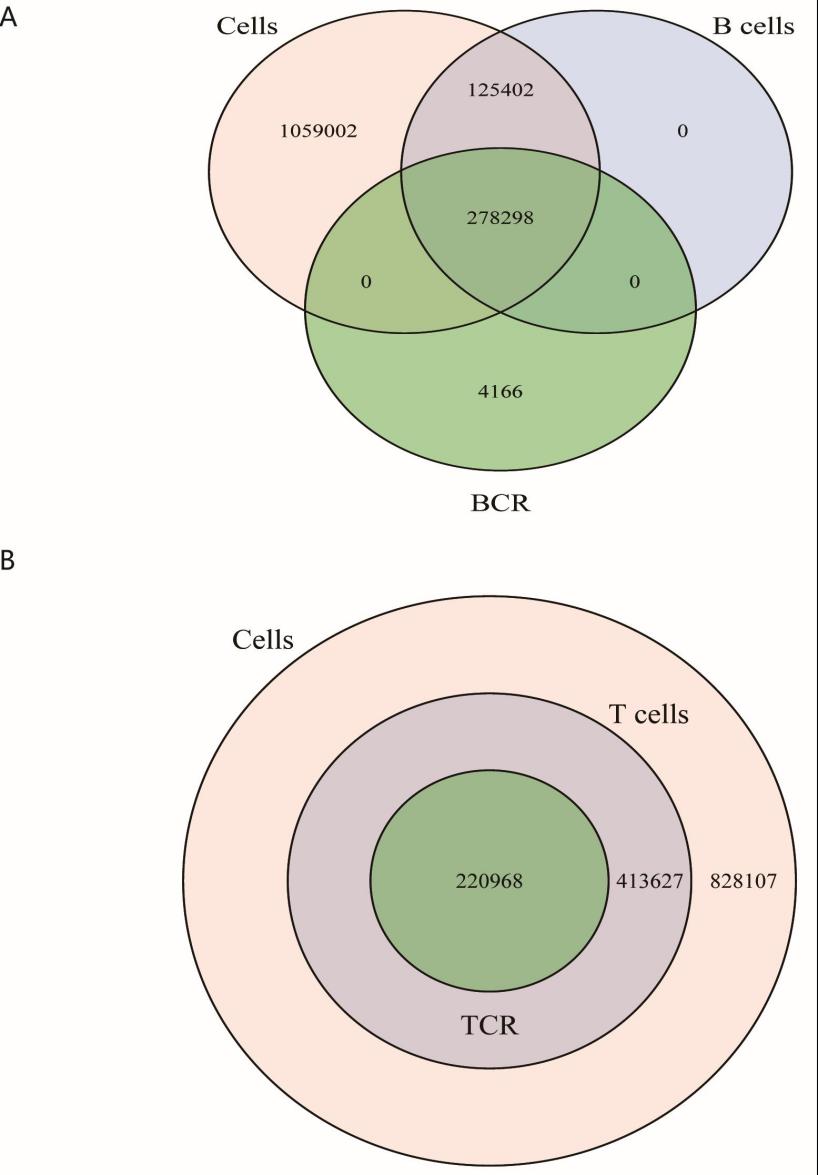


**Supplementary Figure 1. Intersection display of single-cell sequencing data and immune repertoire data.**

1. The number of cells in common between single-cell sequencing data and BCR sequencing data. (B) The number of cells in common between single-cell sequencing data and TCR sequencing data.


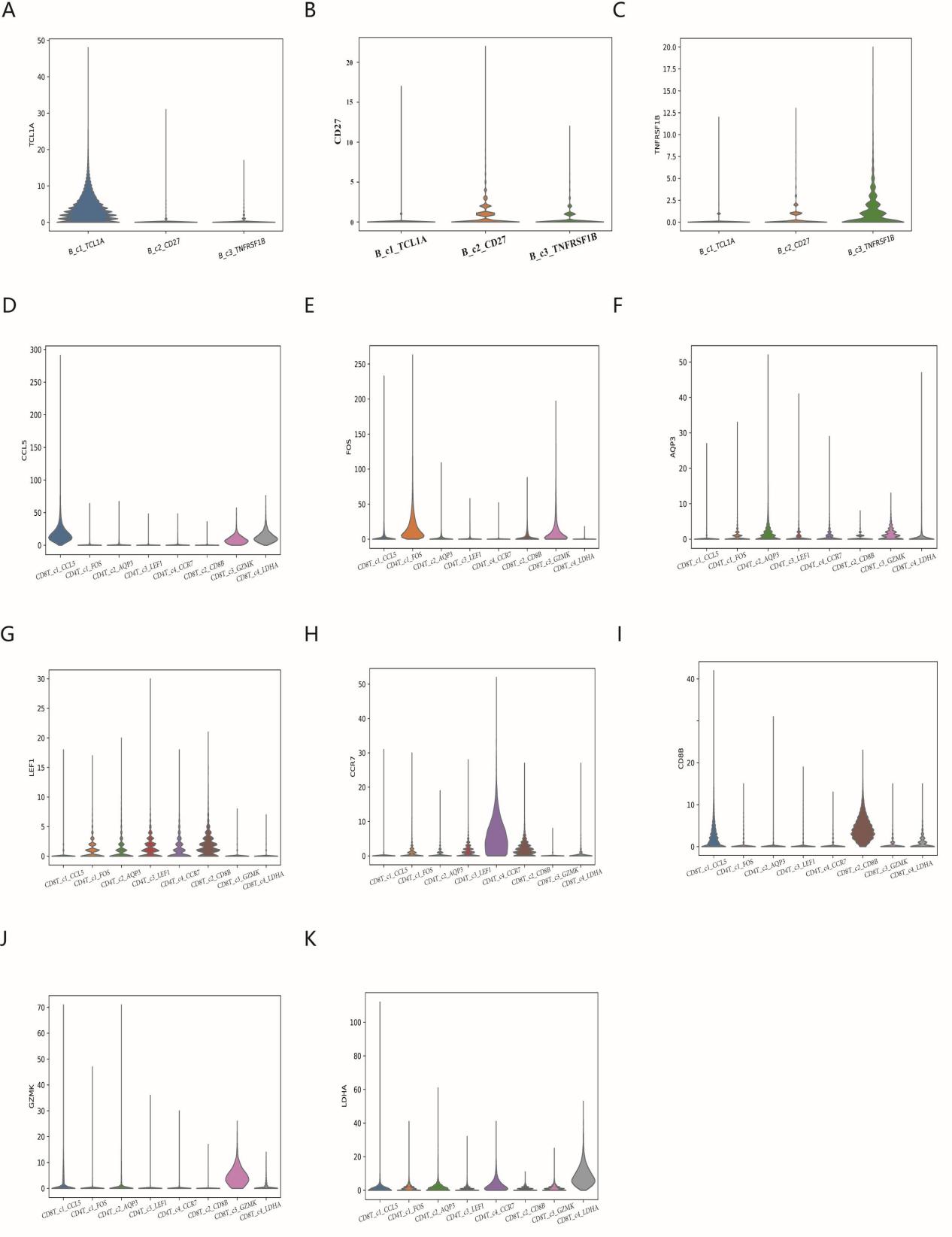


**Supplementary Figure 2. Display of marker genes of cell subpopulations.**

1. The expression of TCL1A in B cell populations. (B) The expression of CD27 in B cell populations. (C) The expression of TNFRSF1B in B cell populations. (D) The expression of CCL5 in T cell populations. (E) The expression of FOS in T cell populations. (F) The expression of AQP3 in T cell populations. (G) The expression of LEF1 in T cell populations. (H) The expression of CCR7 in T cell populations. (I) The expression of CD8B in T cell populations. (J) The expression of GZMK in T cell populations. (K) The expression of LDHA in T cell populations.


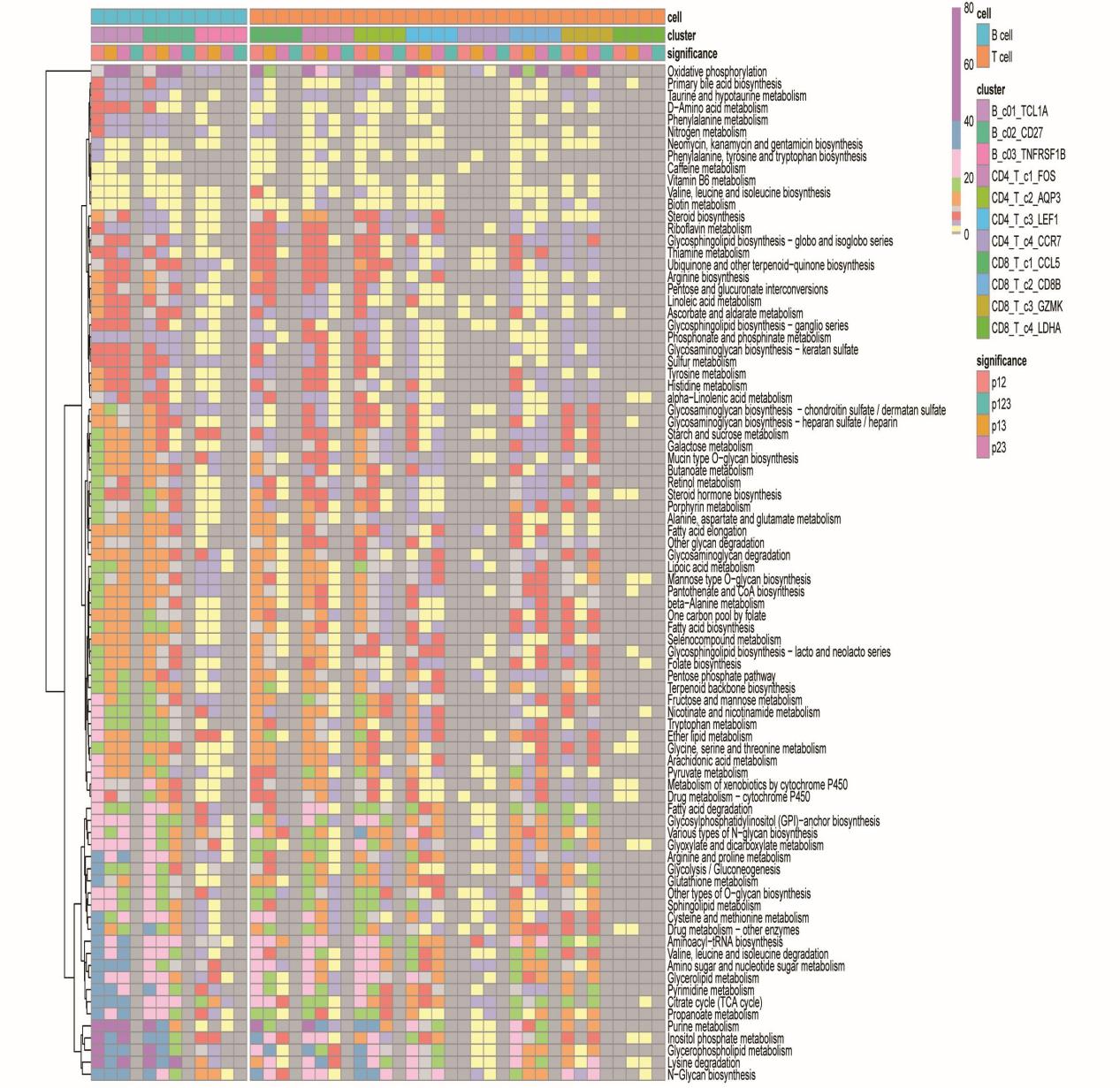


**Supplementary Figure 3. Significance display of metabolic pathways of 11 cell subpopulations.**

Supplementary Table1. the parameters of KDDC framework

| Cell/Sample | Methods | Kmers | Theshold | Anova_pvalue | Features |
| --- | --- | --- | --- | --- | --- |
| B_c1_TCL1A | RF | Kmer3 | 89 | 0.006 | 33 |
| B_c2_CD27 | RF | Kmer2_3_4 | 108 | 0.046 | 307 |
| B_c3_TNFRSF1B | RF | Kmer2_3_4 | 50 | 0.039 | 174 |
| CD8T_c1_CCL5 | DC | Kmer4 | 139 | 0.041 | 87 |
| CD4T_c1_FOS | RF | Kmer4 | 127 | 0.015 | 68 |
| CD4T_c2_AQP3 | MLP | Kmer4 | 127 | 0.026 | 41 |
| CD4T_c3_LEF1 | RF | Kmer4 | 127 | 0.02 | 133 |
| CD4T_c4_CCR7 | MLP | Kmer2_4 | 150 | 0.049 | 434 |
| CD8T_c2_CD8B | SVM | Kmer2_3_4 | 118 | 0.015 | 209 |
| CD8T_c3_GZMK | RF | Kmer2_3 | 121 | 0.012 | 25 |
| CD8T_c4_LDHA | RF | Kmer2 | 150 | 0.049 | 4 |
| Bcr_sample | RF | Kmer3 | 96 | 0.034 | 240 |
| Tcr_sample | RF | Kmer4 | 100 | 0.048 | 128 |
